# Successful Balloon Valvuloplasty in a Case of Neonatal Aortic Critical Stenosis With Bicuspid Aortic Valve (BAV): A Case Report

**DOI:** 10.7759/cureus.68681

**Published:** 2024-09-05

**Authors:** Nimmanagoti Nagaraju, Sagar Karotkar, Vaibhav Raut, Chaitanya Kumar Javvaji, Harshitha Reddy

**Affiliations:** 1 Pediatrics, Jawaharlal Nehru Medical College, Datta Meghe Institute of Higher Education and Research, Wardha, IND; 2 Cardiology, Jawaharlal Nehru Medical College, Datta Meghe Institute of Higher Education and Research, Wardha, IND; 3 Internal Medicine, Jawaharlal Nehru Medical College, Datta Meghe Institute of Higher Education and Research, Wardha, IND

**Keywords:** aortic stenosis, bicuspid aortic valve disease, congenital hear disease, critical aortic stenosis, neonate

## Abstract

A bicuspid aortic valve (BAV) refers to a condition in which the valve has two cusps rather than three. Usually ignored, this manifests itself later in life. This bicuspid valve may manifest earlier in children with significant aortic stenosis because they have a severe left ventricular outflow tract restriction that worsens over time. This syndrome commonly results in congestive heart failure in newborns and early neonatal life. There can be a small amount of significant risk in pediatric patients having this BAV causing stenosis and being one of the reasons for sudden, unexpected death. Morphological differences result from a congenital cardiac abnormality called BAV. This paper emphasizes the importance of a multidisciplinary team in managing BAV and critical aortic stenosis and provides evaluation and treatment guidelines for both conditions. Transcatheter or surgical intervention is used for symptomatic individuals or those with a moderate to severe obstruction of the left ventricular outflow tract.

## Introduction

Congenital heart disease (CHD), which affects 0.8% to 1.2% of live births, is the most common congenital disability [1]. About 3% to 6% of all occurrences of congenital cardiac disease are congenital aortic valve stenosis (AVS), which affects 3.8-4.9 out of every 10,000 live births. Boys are more likely than females to develop this condition, with ratios as high as 3:1 [2]. Aortic valve stenosis can be brought on by birth defects such as bicuspid aortic valves (BAV), double-cusp aortic valves, or valves with fused or deformed cusps [2]. About 20% of cases of congenital AVS are caused by congenital cardiac abnormalities such as patent ductus arteriosus (PDA), ventricular septal defect, or aortic coarctation (COA) [3].

Despite advancements in genetics, the exact etiology and mechanism of congenital aortic valve syndrome in newborns and children remain unknown. Patients with cardiac anomalies (COA, AVS) in the left ventricular outflow tract have been discovered to have disease-causing genetic changes in neurogenic locus notch homolog protein 1 (NOTCH1), which is a single-pass transmembrane receptor. Variations in the suppressor of mothers against decapentaplegic 6 (SMAD6) are also present in patients with BAV and AVS. We discuss a case of aortic valve birth defects and discuss non-surgical possibilities for therapy.

The GATA family of genes (GATA4, GATA5, GATA6) is essential for the development of the heart. Variations in GATA5 have been linked to many forms of congenital cardiac disease, including double outlet right ventricle, ventricular septal defect, atrial septal defect, and tetralogy of Fallot. The BAV is linked to mutations in GATA4, methionine adenosyltransferase 2A (MAT 2A), and other genes may also be involved in BAV [4]. Treatment for valvular aortic stenosis is contingent upon the patient's age, the degree of obstruction, and the suitability of the left heart structure. Treatment options for left ventricle outflow tract obstruction include valve replacement with an emphasis on biventricular repair, judicious aortic valvotomy, and percutaneous inflatable aortic valvoplasty [5]. The Norwood surgery is used to treat individual ventricles. When balloon valvuloplasty is contemplated, the balloon-annulus ratio should be between 0.8 and 1.0, necessitating catheter insertion.

The weakest part of the valve is damaged by balloon inflation, depending on the shape of the valve. While balloon dilation splits the fused commissures in the BAV, it only partially relieves and causes substantial insufficiency in unicuspid valves. Because of the unpredictability of the annulus, aortic disgorging post-swell valvuloplasty can be crucial in infants and children with AVS [6]. Selecting the appropriate balloon catheter is made more accessible by precisely evaluating annular diameters using echocardiography. Cardiac magnetic resonance imaging (MRI) is reliable for assessing left ventricular function, volume, and aortic regurgitation [7]. Speckle-tracking strain echocardiography can be used to quantify left ventricular function in patients who are asymptomatic but have significant mixed lesions [8].

Most patients report a noticeable decrease in the pressure gradient across the aortic valve following balloon aortic valvuloplasty. According to the registry for valvuloplasty and angioplasty for congenital anomalies, transvalvular gradients were reduced by 60% on average, and the treatment-related death rate was 1.9% [9]. High survival rates and different degrees of freedom from surgery and intervention are seen in the mid-term results. Ages under three months and significant post-valvuloplasty gradients are risk factors for restenosis. Although residual blockage is uncommon, long-term follow-up shows that over 25% of patients may progress to restenosis and require reintervention. The severity of early post-valvuloplasty aortic insufficiency indicates the likelihood of later insufficiency. Replacing a failing valve with a prosthetic valve or an allograft can be necessary in situations involving uncorrectable valves, recurrent stenosis, or a tiny aortic annulus with considerable regurgitation [10].

Cardiopulmonary bypass is often used in conjunction with surgical valvotomy [11]. Though it can result in two valve disorders and subsequent surgeries, the Ross technique, which replaces the aortic valve with the pulmonary valve, is frequently performed on children [12] for patients with bicuspid AVS and aortic root dilation over 4.5 cm, combined valve, and aortic root/ascending aorta replacement may be needed. The Bentall procedure is often successful in older adolescents and adults. Surgical mortality has decreased significantly, with improved survival rates [13].

## Case presentation

**Figure 1:**
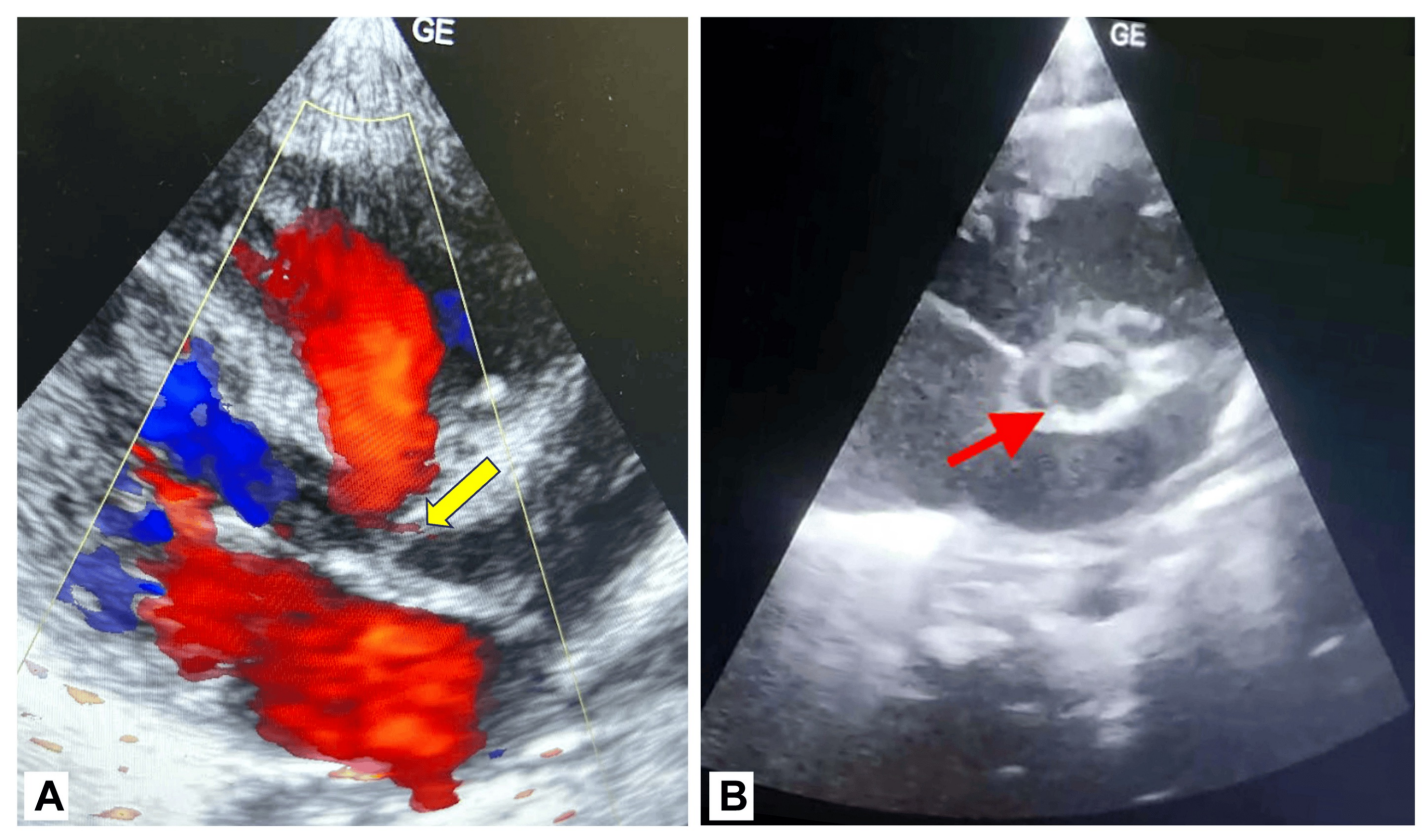
A) Two-dimensional echocardiography (parasternal long axis view) showing aortic stenosis (yellow arrow); B) Two-dimensional echocardiography (short axis view) showing bicuspid aortic valve (red arrow).

A 2.83-kg male baby was delivered by a primipara mother at 39 weeks of pregnancy through a lower cesarean section due to the baby being in a breech position. After birth, the baby cried immediately but then showed slight lower chest retractions and a noticeable grunt, indicating the need for supplemental oxygen. An echocardiogram, or 2D echo, was then performed, which showed the presence of a BAV, severe to moderate aortic stenosis, and a patent foramen ovale with a left-to-right flow pattern (Figure 1). 

The baby was brought to our hospital for a cardiology consultation. The child was admitted to the neonatal intensive care unit. On admission, vitals were a heart rate of 140 beats per minute, respiratory rate of 70 cycles per minute, oxygen saturation of 92%, and capillary refill time of <3 seconds. The child had mild respiratory distress, and other systemic examinations were normal. The child was started on high-flow nasal cannula oxygen therapy and started intravenous antibiotics cefotaxime (50 mg/kg/dose) and amikacin (15 mg/kg/dose). A cardiology consultation was requested, and balloon aortic valvuloplasty was recommended. Under general anesthesia, a high-risk balloon aortic valvuloplasty was done, resulting in substantial blood loss and the need for an intraoperative packed red cell transfusion.

Following surgery, the baby required inotropes due to shock and experienced fever. A blood culture revealed a positive result for *Burkholderia cepacia* that required the administration of intravenous meropenem (40 mg/kg/dose) and tigecycline (1 mg/kg/dose) based on sensitivity. Cerebrospinal fluid examination showed no signs of meningitis, and as the infant gradually improved, room air saturation was maintained. The infant was begun on feeds, which were well tolerated. The baby was discharged with instructions to maintain exclusive breastfeeding for six months and to take medication until further follow-up. Early intervention, kangaroo mother care, and nonnutritive sucking were all recommended. The follow-up plan for the baby involves a regular newborn screening. This case emphasizes the need for prompt therapies and interdisciplinary care while highlighting the successful management of a complicated congenital cardiac condition in a neonate.

## Discussion

A standard aortic root consists of the three aortic sinuses within the Valsalva, divided by commissural gaps and interleaflet triangles. The regions of the handout extend beyond the aortic root's cross-sectional area, supporting its function. The congenital AVS includes the single-cusp, double-cusp, triple-cusp, and quadruple-cusp forms, with BAV affecting about 2% of the population [1]. The classification of BAV is determined by the number of raphe lines, categorizing it into three types: type 0 (no raphe), type 1 (one raphe), and type 2 (two raphe). Type 1 is the most common, making up nearly 90% of BAV cases. Stenosis happens when the aortic valve leaflets are more restricted.

Then, sinus connections, leaflets are twisted, or commissures are combined. A BAV usually has a poor prognosis with age. A BAV is an autosomal-dominant condition with varied inheritance patterns, probably due to abnormal neural crest cell migration [3]. Although AVS development is nearly complete by the first trimester, it can continue throughout pregnancy, with severe cases occurring by the second trimester, indicating that the left ventricle and aortic root are underdeveloped. The right ventricle usually compensates for lower left ventricular output dysfunction in isolated AVS. When the ductus arteriosus closes, systemic cardiac output drops, which can lead to heart failure in symptomatic newborns and infants. An AVS initially compensates for pressure overload in children and adolescents by increasing wall stress and left ventricular mass. As stenosis advances, left ventricular hypertrophy causes diastolic dysfunction and expanded myocardial oxygen utilization, prompting myocardial ischemia and fibrosis.

Diastolic dysfunction makes it more critical for the atrial contribution to fill the left ventricle, which could cause heart failure [2,3]. A normal-sized BAV with some commissural fusion, which responds well to balloon dilation, is the most common abnormality in mild aortic stenosis [7-9]. In cardiac catheterization, balloon dilation valvuloplasty can be performed on young babies through the umbilical artery without open-heart surgery. However, open-heart surgery is necessary for more intricate valve problems, such as severe hardening of the valve or underdeveloped valve rings that necessitate the surgical replacement of the aortic valve. The Ross operation, which is appropriate for children, involves replacing the aortic valve with the pulmonary valve and is considered better than a mechanical valve that demands lifelong use of anticoagulant drugs. For small aortic valves, more elaborate surgeries like the Konno procedure may be considered, which widens the aortic valve ring to accommodate a more appropriately sized prosthetic valve or pulmonary valve autotransplant. The aortic valve reconstruction surgery, or Ozaki, procedure has excellent short-term results and should be considered for valve reconstruction in pediatric patients with congenital aortic and truncal valve disease. Longer-term follow-up is necessary to determine the optimal patch material and late valve function.

## Conclusions

Given that the simultaneous presence of BAV and severe aortic stenosis in a newborn is rare, it's vital for pediatricians to suspect the condition with a strong level of awareness. This case highlights the importance of early identification and intervention for effectively managing such conditions. A key diagnostic tool for this is 2D echocardiography. A team of specialists, including cardiologists, cardiothoracic surgeons, and neonatologists, is required to care for a newborn with these conditions. Prompt diagnosis and treatment, whether through surgery or less invasive procedures, can lead to better outcomes. To address potential long-term complications associated with BAV and aortic stenosis, as well as to ensure the healthy growth and development of affected newborns, regular follow-up and monitoring are essential.
